# Obstructive sleep apnea is underrecognized and underdiagnosed in patients undergoing bariatric surgery

**DOI:** 10.1007/s00405-012-1948-0

**Published:** 2012-02-05

**Authors:** M. J. L. Ravesloot, J. P. van Maanen, A. A. J. Hilgevoord, B. A. van Wagensveld, N. de Vries

**Affiliations:** 1 Department of Otolaryngology/Head and Neck Surgery, St. Lucas Andreas Hospital, Jan Tooropstraat 164, 1061 Amsterdam, The Netherlands; 2 Department of Clinical Neurophysiology, St. Lucas Andreas Hospital, Jan Tooropstraat 164, 1061 Amsterdam, The Netherlands; 3 Department of Surgery, St. Lucas Andreas Hospital, Jan Tooropstraat 164, 1061 Amsterdam, The Netherlands

**Keywords:** Obstructive sleep apnea, Bariatric surgery, Obesity, Body mass index, Neck, Polysomnography

## Abstract

The aim of this study was to evaluate prevalence of obstructive sleep apnea among patients undergoing bariatric surgery and the predictive value of various clinical parameters: body mass index (BMI), neck circumference (NC) and the Epworth Sleepiness Scale (ESS). We performed a prospective, multidisciplinary, single-center observational study including all patients on the waiting list for bariatric surgery between June 2009 and June 2010, irrespective of history or clinical findings. Patients visited our ENT outpatient clinic for patient history, ENT and general examination and underwent a full night polysomnography, unless performed previously. As much as 69.9% of the patients fulfilled the criteria for OSA (mean BMI 44.2 ± SD 6.4 kg/m^2^); 40.4% of the patients met the criteria for severe OSA. The regression models found BMI to be the best clinical predictor, while the ROC curve found the NC to be the most accurate predictor of the presence of OSA. The discrepancy of the results and the poor statistical power suggest that all three clinical parameters are inadequate predictors of OSA. In conclusion, in this large patient series, 69.9% of patients undergoing BS meet the criteria for OSA. More than 40% of these patients have severe OSA. A mere 13.3% of the patients were diagnosed with OSA before being placed on the waiting list for BS. On statistical analysis, increased neck circumference, BMI and the ESS were found to be insufficient predictors of the presence of OSA. Polysomnography is an essential component of the preoperative workup of patients undergoing BS. When OSA is found, specific perioperative measures are indicated.

## Introduction

Obesity, declared a global epidemic by the World Health Organization, is associated with a number of illnesses such as diabetes, cardiovascular disease and cancer (http://www.who.int) [1]. Equally, obesity is a significant risk factor for obstructive sleep apnea (OSA), the most prevalent sleep disordered breathing problem. OSA affects 2–26% of the general population, depending on gender, age and definition of the used criteria [2, 3]. To make matters worse, obesity is dramatically on the rise. In 2005, 400 million adults worldwide were obese. The WHO projects that in 2015, 700 million adults will be obese (http://www.who.int).

The exact pathophysiology of OSA in obese patients remains poorly understood, but it is thought that in obese patients the local fatty tissue deposition in the neck results in reduction of the lumen of the upper airway, thereby reducing airflow and inducing airway collapse [4].

In patients with morbid obesity, who have failed conservative treatment, bariatric surgery (BS) can be considered. BS is not only the most effective, long-term treatment modality in these patients for losing weight, but is also known to have a positive effect on comorbidities. It is therefore becoming increasingly popular. The benefits of bariatric surgery are increasingly reported, but concern about the safety is also heightened [5].

Patients with OSA are particularly vulnerable during anesthesia and sedation and at an increased risk of developing respiratory and cardiopulmonary complications postoperatively [6]. These risks can be decreased by adequate management of the OSA. Furthermore, a recent study by the Longitudinal Assessment of Bariatric Surgery group (LABS) shows that a history of OSA is significantly associated with an increased risk of major perisurgical adverse outcomes in patients undergoing BS [5]. Additionally, OSA was found to triple the risk of perioperative death in a recent single-surgeon report of 1000 Roux-en-Y gastric bypass procedures [7].

Anesthetist and surgeons should be aware that undiagnosed OSA is common; OSA remains undiagnosed in an estimated 93% of women and 82% of men [6, 8]. This might hold true even more in the BS population.

Bearing this in mind, and with the aim of preventing OSA-related complications of BS, we were interested to see which percentage of patients undergoing bariatric surgery in our clinic had OSA. Secondly, polysomnographies (PSG) are time consuming, costly and patient burdensome. We were interested in measuring the predictive value of various clinical parameters: body mass index (BMI), neck circumference (NC) and Epworth Sleepiness Scale (ESS).

## Methods

### Patients

We performed a prospective, multidisciplinary, single-center observational study involving a consecutive series of patients being evaluated for BS in our clinic from June 2009 until June 2010. Data collection for this study was approved by the institution’s ethics committee. Patients meeting the International Federation for the Surgery of Obesity (IFSO) (http://www.ifso.com) criteria were eligible for BS, specifically patients aged 18–65 years, with a BMI > 40 kg/m^2^ or BMI > 35 kg/m^2^ with associated comorbidity (e.g., hypertension, diabetes, OSA or joint problems). Secondly, patients were required to have made sufficient attempts at weight loss using conservative measures and must be motivated for dietary and behavior modification. There was flexibility in these guidelines. Some patients with a BMI < 35 kg/m^2^ were also included if comorbid disease was present. A few exceptions were also made concerning the age restriction. Participants with a previous diagnosis of OSA were not excluded from our analysis. Various BS types are performed in our clinic: laparoscopic gastric banding, Swedish type of adjustable gastric banding (SAGB), laparoscopic gastric bypass and gastric sleeve resection. All patients eligible for BS underwent a mandatory preoperative screening for OSA in addition to our routine preoperative workup. If the AHI was greater than 15/h, CPAP was prescribed.

Apart from patients with OSA previously diagnosed elsewhere, preoperatively all patients on the waiting list for BS visited the ENT outpatient department. Information was gained using patient history, ENT and general examination and a full overnight polysomnography (PSG). Weight, length (BMI) and NC at the level of the cricothyroid membrane were measured. The following BMI grading system was implemented: obese (BMI 30–34.9 kg/m^2^), severely obese (BMI 35–39.9 kg/m^2^), morbidly obese (BMI 40–49.9 kg/m^2^) and super obese (BMI > 50 kg/m^2^) http://www.ifso.com. The patients completed a questionnaire containing various questions concerning possible daily or nocturnal symptoms, intoxications, medication and medical history. The ESS was included in the questionnaire. Patients scored themselves on a scale of 0–3 on how easily they would fall asleep in eight different situations, giving an overall score between 0 and 24; the higher the score, the sleepier was the individual [9].

### Polysomnography

Besides patients with OSA previously diagnosed elsewhere, all patients underwent a full night comprehensive sleep study using a digital Embla recorder (Flaga Medical devices, Reykjavik, Iceland). This records the sleep architecture (derived from electroencephalogram, electrooculogram and submental electromyogram), respiration (thoracic and abdominal measurement), movements of limbs, nasal airflow and the intensity of the snoring (the last two measured by pressure sensor). Transcutaneous pulse oximetry was used to monitor oxygen saturation (SaO_2_) and heart rate [10].

The severity of OSAS is expressed in the apnea hypopnea index (AHI). Obstructive apneas were defined as cessation of airflow for at least 10s. Hypopneas were defined as periods of reduction of >30% oronasal airflow for at least 10s and a ≥4% decrease in oxygen saturation. Arousals were not scored as hypopneas. The apnea/hypopnea index (AHI) was calculated as the sum of total events (apneas and hypopneas) per hour of sleep. An AHI of 5–15/h is mild OSAS, an AHI of 15–30/h is moderate and AHI > 30/h is severe OSAS, as assessed by polysomnography [2, 10].

### Statistical analysis

Statistical analysis was performed using Microsoft Excel and SPSS statistical software (version 18, SPSS Inc., Chicago, USA).

The distribution of recorded variables was characterized by calculating the mean and standard deviation. Since some parameters (especially the AHI) were expected to be non-normally distributed, also the median and range were calculated. Data are given for both the total study population and subdivided for women and men. The results of women and men were compared using an unpaired *t* test, with additional non-parametric Mann–Whitney when applicable. Differences were considered significant when *p* < 0.05.

The prevalence of OSA and OSA severity was subdivided for obesity severity subgroups. The relation between the AHI and patient characteristics was further evaluated employing stepwise linear regression and binomial logistic regression. A *p* value of <0.05 was considered to be statistically significant.

The sensitivity and specificity of the clinical predictor variables for the presence versus absence of OSA (AHI > 5/h) and moderate to severe OSA (AHI > 15/h) were used to plot receiver operating characteristic (ROC) curves.

## Results

A total of 289 consecutive patients were recruited. Ten patients did not show up for their ENT outpatient clinic and PSG appointment. Of the remaining 279 patients, 214 (76.7%) were women and 65 (23.3%) men. Patient baseline characteristics are shown in Table 1.

**Table 1:** Patient characteristics: clinical and polysomnographic parameters

Patient characteristics	Mean	Median	Range
Women	214 (76.7%)	–	–
Men	65 (23.3%)	–	–
Age (years)	45.1 ± 10.6	46.0	(17–67)
BMI (kg/m^2^)	44.2 ± 6.4	42.8	(33–66)
Neck circumference (cm)	42.6 ± 4.8	42.0	(34–59.8)
ESS	4.3 ± 3.8	3.0	(0–17)
AHI (per h)	23.9 ± 27.7	12.4	(0–142)
AI	11 ± 21.4	1.6	(0–127)
Arousal index (per h)	7.5 ± 8.4	5	(0–54.7)
Mean SaO_2_ (%)	93.8 ± 3.3	94.7	(74–99)
Minimum SaO_2_ (%)	80.8 ± 10.7	83.0	(50–95)
Desaturation index (DI)	16.3 ± 23.4	5.3	(0–106)

± indicates standard deviation

*AI* apnea index; *AHI* apnea hypopnea index; *BMI* body mass index, *ESS* Epworth Sleepiness Scale; *OSA* obstructive sleep apnea; *SaO*_2_ oxygen saturation

An unpaired *t* test was conducted to compare baseline characteristics in men and women. There was a significant difference in the AHI (*p* < 0.0003), AI (*p* < 0.0004), Arousal index (*p* < 0.006), DI (*p* < 0.0003), mean O_2_ saturation (*p* = 0.001), minimum O_2_ saturation (*p* < 0.0007) and NC (*p* < 0.0003) between men and women.

In our study population, men were found to have a higher AHI, AI, arousal index and DI, and lower mean and minimum O_2_ saturation (Table 2). Therefore, the male and female study population should be analyzed independently. Application of a non-parametric test provided no new insights.

**Table 2:** Patient characteristics subdivided for women and men

Patient characteristics	Men	Women
Age (years)	48.5 ± 9.3	44.0 ± 10.8
BMI (kg/m^2^)	44.3 ± 7.1	44.2 ± 6.2
Neck circumference (cm)	48.0 ± 3.9	41.2 ± 4.0
ESS	5.0 ± 4.2	4.2 ± 3.6
AHI (h^−1^)	45.9 ± 29.9	17.3 ± 23.3
AI (h^−1^)	25.7 ± 27.1	7.0 ± 17.5
Arousal index (h^−1^)	10.6 ± 8.9	6.7 ± 8.1
Mean SaO_2_ (%)	92.3 ± 3.0	94.1 ± 3.4
Minimum SaO_2_ (%)	74.6 ± 11.3	82.5 ± 9.9
Desaturation index (DI)	32.2 ± 26.8	11.8 ± 20.3

± indicates standard deviation

*AI* apnea index; *AHI* apnea hypopnea index; *BMI* body mass index, *ESS* Epworth Sleepiness Scale; *OSA* obstructive sleep apnea; *SaO*_2_ oxygen saturation

**Fig. 1:**
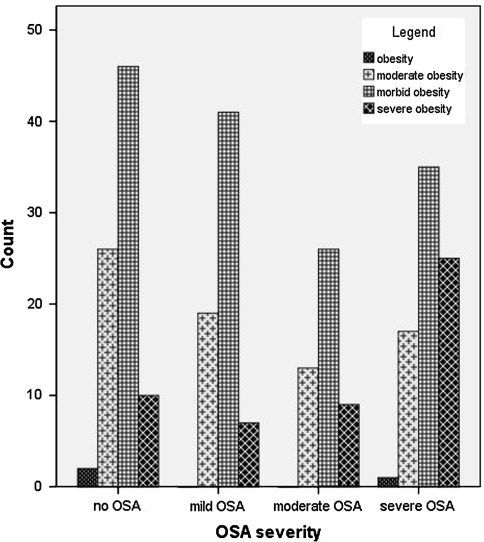
Severity OSA stratified by BMI

Three (1.1%) of the patients were obese, 75 (26.9%) severely obese, 149 (53.6%) morbidly obese, 51 (18.3%) super obese. OSA stratified by BMI and the severity of OSA by BMI are depicted in Table 3 and Fig. 1, respectively. A total of 112 (40.1%) patients underwent or are on the waiting list for SAGB (average BMI 41 ± SD 4 kg/m^2^), 155 (55.6%) laparoscopic gastric bypass (average BMI 46.1 ± SD 6.7 kg/m^2^) and 12 (4.3%) gastric sleeve surgery (average BMI 49.4 ± SD 8.5 kg/m^2^). A mere 37 (13.3%) patients had been previously diagnosed with OSAS (AHI: 42.5 ± SD 27.2 per h) in our hospital (12 pts) or elsewhere (25 pts) before being placed on the waiting list for BS. Based on the PSG results, 195 (69.9%) patients were diagnosed with OSA, specifically 67 (34.7%) with mild OSA, 48 (24.9%) with moderate OSA and 78 (40.4%) with severe OSA. 69.2% of the patients diagnosed with OSA were female and 30.7% male.

**Table 3:** Number of patients with OSA stratified by BMI

OSA stratified by BMI	OSA	No OSA	Total no.	OSA (%)
Obese (30–34.9 kg/m^2^)	1	2	3	33.3
Severely obese (35–39.9 kg/m^2^)	49	26	75	65.3
Morbidly obese (40–49.9 kg/m^2^)	103	46	149	69.1
Super obese (>50 kg/m^2^)	41	10	51	80.4

*BMI* body mass index, *OSA* obstructive sleep apnea

Stepwise linear regression was performed with AHI as the dependent variable. Independent variables evaluated were the BMI, NC and ESS [adjusted *R* ^2^ = 0.236; *F* = 22.9, *p* < 0.0001 (using the stepwise method)]. In men, only the BMI was associated with the AHI (adjusted *R* ^2^ = 0.167; *F* = 10.2, *p* = 0.003). Addition of the NC and ESS gave no significant improvement of the model. In women, the BMI also had a strong predictive value. Addition of NC made significant improvement to the model, but ESS did not (adjusted *R* ^2^ = 0.113; *F* = 11.5, *p* < 0.0001).

The AHI data are not strictly normally distributed. However, also after normalizing the data with a square root transformation, there was no improvement in the association between NC, ESS and the dependent variable (AHI).

Binomial logistic regression was used to identify independent variables associated with the presence or absence of (1) an AHI greater than 5/h or (2) 15/h.

Results showed that in women, the BMI was the only significant predictor of an AHI greater than 5/h (odds ratio [OR] = 1.072, *p* = 0.018, 95% CI 1.012–1.135) and of an AHI greater than 15/h ([OR] = 1.102, *p* = 0.001, 95% CI 1.042–1.165). In men, NC was a significant predictor of an AHI greater than 15/h ([OR] = 1.278, *p* = 0.026, 95% CI 1.030–1.586). No significant predictor was found for AHI greater than 5/h.

The results of the ROC curves were disappointing; of all three clinical parameters, no cutoff values were found to have both a sensible sensitivity (>0.8) and a useful specificity (>0.9). No difference was seen between men and women.

**Fig. 2:**
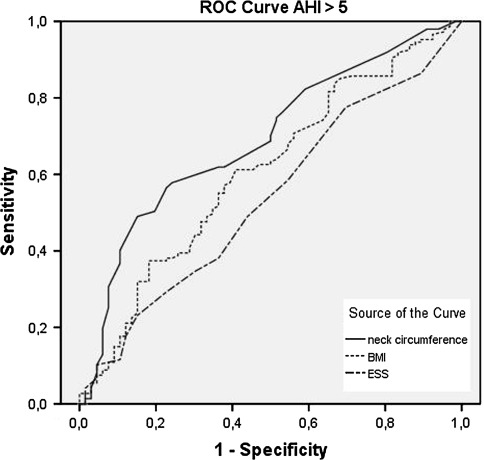
ROC curve comparing sensitivity and specificity of neck circumference (*NC*), body mass index (*BMI*) and Epworth Sleepiness Scale (*ESS*) of an AHI > 5/h. The mean area under the curve (AUC) for NC, BMI and ESS were 0.69 ± 0.4 (95% CI 0.62–0.77), 0.61 ± 0.4 (95% CI 0.53–0.69), 0.54 ± 0.4 (95% CI 0.45–0.62), respectively. No cutoff values were found to have both a sensible sensitivity (>0.8) and a useful specificity (>0.9)

**Fig. 3:**
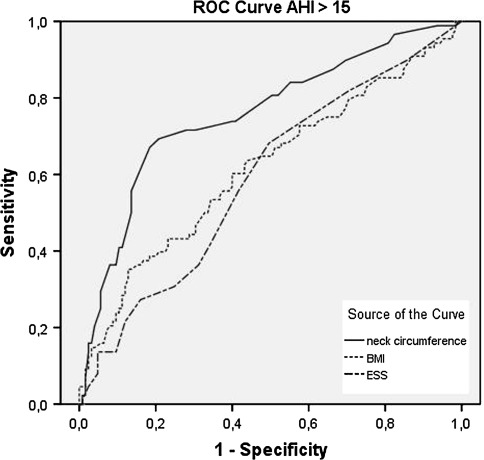
ROC curve comparing sensitivity and specificity of neck circumference (*NC*), body mass index (*BMI*) and Epworth Sleepiness Scale (*ESS*) of an AHI > 15/h. The mean area under the curve (AUC) for NC, BMI and ESS were 0.76 ± 0.4 (95% CI 0.69–0.82), 0.62 ± 0.4 (95% CI 0.54–0.69) and 0.59 ± 0.4 (95% CI 0.51–0.67), respectively. No cutoff values were found to have both a sensible sensitivity (>0.8) and a useful specificity (>0.9)

The neck circumference was found to be the most accurate predictor of the presence of OSA when the AHI as greater than 5/h (Fig. 2). The same was found when predicting an AHI greater than 15/h (Fig. 3).

## Discussion

In this series of consecutive patients undergoing BS, we found a 69.9% prevalence of OSA. More than 40% of these patients were diagnosed with severe OSA. Of the 195 patients diagnosed with OSA, 69.2% were female: a 1:2.3 ratio (♂:♀), which is opposite to the typical OSA male female ratio of 2:1 (♂:♀) [2]. The raised percentage of women with OSA is caused by a skewed gender distribution within our study population. More than three-quarters of our study population was female; comparative to earlier reports that women seek surgical weight loss treatment nearly four times more often than men [11].

Unlike many other studies, we used no selective inclusion criteria such as the ESS as a screening tool. Irrespective of history or clinical findings, all patients being evaluated for BS underwent a polysomnography, unless performed previously.

Our results are consistent with similar studies (see Table 3). Using synonyms for: bariatric surgery, obstructive sleep apnea and polysomnography, an online systematic search was performed of the MEDLINE and EMBASE database on the 4th April 2011. Ten relevant articles were found. In each study, all patients being evaluated for bariatric surgery underwent a polysomnography as part of routine screening for OSA regardless of symptoms and without use of screening tools such as the ESS.

Four articles were omitted from our overview (see Table 4), owing to various applied inclusion criteria (a BMI ≥ 40 kg/m^2^ or Asian race) and articles, which did not apply the AASM OSA guidelines [12–15].

**Table 4:** Outcomes of similar studies, in which all patients being evaluated for bariatric surgery underwent a polysomnography, irrespective of history or clinical findings

Reference	Total *n*	OSA *n*	Prevalence OSA (%)	Severe OSA (%)	Mean AHI (h^−1^)	Mean BMI (kg/m^2^)
Frey et al. [19]	41	29	71.0	21.0	23.0	47.0
O’Keeffe et al. [18]	170	131	77.0	23.7	–	–
Lopez et al. [17]	290	227	78.0	38.3	–	52.0
Hallowell et al. [21]	249	227	91.0	–	–	49.0
Sareli et al. [16]	342	264	77.2	27.2	24.9	49.5
Lee et al. [20]	176	126	71.6	48.0	28.0	42.0
All	1268	1004	79.2	33.1^a^	25.7^b^	48.8^c^

*AHI* apnea hypopnea index, *BMI* body mass index, *OSA* obstructive sleep apnea

^a^Only studies presenting percentage severe OSA data

^b^Only studies presenting mean AHI data

^c^Only studies presenting mean BMI data

To our knowledge, following Sareli et al. and Lopez et al. [16, 17], our group researched the third largest study population. Together with O’Keeffe et al. [18], the above-mentioned authors report a prevalence of 77–78%. Our results are more in line with the results of Frey et al. and Lee et al., but it should be noted that Lee et al. studied a predominantly Asian population [19, 20]. Hallowell stands out with a staggering 91% prevalence [21].

Several studies reported that the prevalence of OSA increased as the BMI increased, which may explain why our prevalence was lowest of all [17, 18]. In contrast, we measured a high percentage of patients with severe OSA.

We have two main study limitations, the first being that OSA was an inclusion criterion for bariatric surgery, in accordance with the IFSO guidelines: a potential bias, which could result in an overestimation of the prevalence of OSA in our bariatric surgery population. A mere 13.3% (37) patients were diagnosed with OSA before being placed on the waiting list for BS; 62.2% of these patients had a BMI greater than 40 kg/m^2^ and were therefore eligible for BS regardless of the presence of OSA. Four of the remaining patients with a BMI smaller than 40 kg/m^2^, had no other comorbid disease than OSA. The rest also suffered from hypertension, diabetes or had joint problems. As this group was minimal, we chose to include these patients in the series so as to avoid underestimating the prevalence of OSA in our bariatric surgery population.

The second limitation of the study is absent data, in particular from patients who had previously been diagnosed with OSA elsewhere. These patients did not visit our outpatient department, consequently patient information such as ESS or NC was unavailable. We also had limited access to their specific PSG data. ESS data were available for 78.5% of the patients, and NC measurements for 82.4%.

ESS is considered a useful screening tool for OSA in the adult population; but as reported by Sareli et al. [16], in the bariatric population, ESS cannot independently reliably predict the presence of OSA. Our data support this observation; we found ESS not to be significantly related to the presence of OSA in patients undergoing BS. Hence, ESS is not a reliable predictor of OSA in this patient population, despite often being used in BS centers as a screening tool [21].

We used various statistical techniques to analyze the data. The various regression models and the ROC curves give discrepant results, mainly due to the non-normal distribution of the data and data values of zero or close to zero.

The poor statistical power and discrepancy of the results strengthen our defense. We contend that all three clinical parameters are inadequate predictors of OSA and that PSG is an essential component of the preoperative workup of patients undergoing BS. Despite the high test probability of moderate to severe OSA in obese patients, the high costs and patient burden of PSGs as well as the increasing prevalence of obesity and BS, the Task Force of the AASM does not advise the use of unattended portable monitoring (PM) for general screening, as there is yet insufficient evidence to guide the use of PM [22].

A recent, unique study by Hallowell et al. compared a series of consecutive patients who underwent mandatory OSA evaluation with a second group who were selected for a preoperative sleep study based on clinical suspicion and a raised ESS in preparation for the bariatric surgery program. The authors suggest that OSA is grossly underdiagnosed in the bariatric population and concludes that clinical evaluation including the ESS is inadequate to identify the true prevalence of OSA [20].

We found that a substantial number of patients, even patients with extremely high AHIs, were completely unaware of their OSA. A mere 13.3% of the patients were diagnosed with OSA before being placed on the waiting list for BS; 37 (13.3%) patients had an AHI > 60/h and only 11 patients were aware of their extreme OSA. Patients are often asymptomatic or relate complaints of fatigue and hypersomnolence to their obesity and/or other comorbidities (e.g., diabetes) and do not realize that these are actually OSA related. More often than in the general population, these patients sleep/live alone and a history of a bed partner is often lacking, which collaborates with patients being often unaware of their breathing abnormalities during sleep.

The finding of OSA may in fact influence the indication for BS being a BMI > 40 kg/m^2^ in patients without comorbidity, or BMI > 35 kg/m^2^ with comorbidity. Therefore, in otherwise healthy patients with a BMI between 35 kg/m^2^ and 40 kg/m^2^, the finding of OSA widens the indication for BS.

The finding of OSA has important perioperative implications. Patients with OSA have been shown to have increased preoperative risk and specific perioperative measures have to been taken [4, 23].

All patients with moderate to severe OSA should have perioperative CPAP therapy [23]. To what extent perioperative CPAP therapy should also be applied in mild OSA remains to be elucidated.

Intubation might be difficult, and specific methods of intubation can be indicated. In case intubation is impossible and a tracheostomy must be performed, longer than usual tracheal cannulas might be necessary [6]. Postoperatively, the use of morphinomimetic painkillers and other muscle tone reducing medication is contraindicated in patients with OSA, or can only be used with postoperative monitoring [23].

## Conclusion

We conclude that 69.9% of patients undergoing BS meet the criteria for OSA. More than 40% of these patients have severe OSA. Increased neck circumference, BMI or ESS cannot reliably predict the presence of OSA. Polysomnography is an essential component of the preoperative workup of patients undergoing BS. When OSA is found, specific perioperative measures are indicated. We are currently following these patients and aim to publish a paper showing the results of postoperative PSG results shortly.

## Abbreviations

AASM: American Academy of Sleep Medicine
AHI: Apnea hypopnea index
AI: Apnea index
BMI: Body mass index
BS: Bariatric surgery
CPAP: Continuous positive airway pressure
DI: Desaturation index
ENT: Ear nose and throat
ESS: Epworth Sleepiness Scale
h: Hour
IFSO: International Federation for the Surgery of Obesity
kg: Kilogram
m: Meter
OSA: Obstructive sleep apnea
PSG: Polysomnography
ROC: Receiver operating characteristic
SAGB: Swedish adjustable gastric banding
SaO_2_: Oxygen saturation
WHO: World Health Organization
